# Evaluation of Death among the Patients Undergoing Permanent Pacemaker Implantation: A Competing Risks Analysis

**Published:** 2017-06

**Authors:** Haleh GHAEM, Mohammad GHORBANI, Samira ZARE DORNIANI

**Affiliations:** 1. Research Center for Health Sciences, Institute of Health, Dept. of Epidemiology, School of Health, Shiraz University of Medical Sciences, Shiraz, Iran; 2. Research Committee, Shiraz University of Medical Sciences, Shiraz, Iran; 3. Anesthesiology and Critical Care Research Center, Shiraz University of Medical Sciences, Shiraz, Iran

**Keywords:** Pacemaker, Competing for risk, Sick sinus syndrome

## Abstract

**Background:**

Permanent artificial pacemaker is one of the important therapies for treatment of cardiac conduction system problems. The present study aimed to determine the association between some predictive variables and all-cause and cause-specific mortality in the patients who had undergone pacemaker implantation.

**Methods:**

This study was conducted on 1207 patients who had undergone permanent pacemaker implantation in the hospitals affiliated with Shiraz University of Medical Sciences, Iran, from Mar 2002 to Mar 2012. The variables that existed in the patients’ medical records included sex, diabetes mellitus, obesity, cerebrovascular accident, cardiomegaly, smoking, hypertension, ischemic heart disease, congenital heart disease, sick sinus syndrome, and atrial fibrillation. Competing risks model was used to assess the association between the predictive variables and cause-specific (i.e., cardiac and vascular) mortality.

**Results:**

The patients’ mean age was 66.32±17.92 yr (70.62±14.45 yr in the patients with single-chamber pacemakers vs. 61.91±17.69 yr in those with two-chamber pacemakers) (*P*<0.001). Sick sinus syndrome and age increased the risk of all-cause mortality, while two-chamber pacemaker decreased this risk. Obesity increased the risk of cardiac death, and diabetes mellitus and heart valve disease increased the risk of vascular death.

**Conclusion:**

The variables predicting mortality in all-cause model were completely different from those in cause-specific model. Moreover, death in such patients may occur due to reasons other than pacemaker. Therefore, future studies, particularly prospective ones, are recommended to use competing risks models.

## Introduction

Using permanent artificial pacemaker is one of the important therapies for treatment of cardiac conduction system problems (1). The first artificial pacemaker was implanted about 60 yr ago and more than 400 thousand pacemakers are implanted for patients around the world each year. Today, with advancement of technology, very advanced pacemakers are available (2). Progress in pacemaker technology in the past decade indicates the necessity to update pre-implantation determinants of patient’s prognosis (3).

Long-term survival after implantation is one of the important issues in evaluation and selection of a permanent artificial pacemaker. For time to event data, Kaplan-Meier survival analysis methods are usually employed. However, patients with permanent artificial pacemakers may die for reasons other than the pacemaker. Therefore, there is a competing risks situation where Kaplan-Meier survival analysis is not appropriate. Most of the studies assessed the association between pacemaker mode and cause-specific mortality, have failed to consider the “competing risks” of other causes of death (4–8). When there are no or low competing risks, Cox regression model is suitable to be used. However, in case of high competing risks, especially in the elderly patients (9), this method may overestimate the absolute risk of the event of interest. Cox method assumes that the cases that die and censored because of competing risks can experience the event of interest in future, which is wrong (10). Moreover, when there is a competing risks situation, survival methods cannot accurately predict the probability of survival rate (11–15). Thus, using competing risks is suitable in diseases, such as heart disease and cancers where there are multiple failure types, because it can estimate the impact of exposure to different causes of death accurately (16). No studies have been conducted on the relationship between pacemaker mode and its changes and cause-specific mortality using competing risks models.

Therefore, the present study aimed to determine the association between pacemaker mode and all-cause and cause-specific mortality in the patients who had undergone pacemaker implantation in the hospitals affiliated with Shiraz University of Medical Sciences, Iran, from Mar 2002 to Mar 2012.

## Methods

This study was conducted on 1207 patients who had undergone permanent pacemaker implantation. The data were collected from the patients’ medical records. The variables that existed in the medical records included sex, diabetes mellitus, cerebrovascular accident, obesity, smoking, cardiomegaly, hypertension, congenital heart disease, ischemic heart disease, sick sinus syndrome and atrial fibrillation. The patients’ survival was determined by phone contact. Additionally, the leading cause of the patients’ death was extracted from the registration system of the Department of Health of Shiraz University of Medical Sciences, Iran.

Competing risks situation arises when an individual experiences more than one type of event and occurrence of an event (death from vascular disease) prevents the occurrence of another event (death from cardiac disease) (17, 18). When there are competing risks situations, Kaplan-Meier estimation cannot be interpreted as a probability; therefore, a specific approach is required based on the cumulative incidence function (19). Competing risks regression models allow us to identify independent risk factors for two events (death from cardiac and vascular diseases) and to create two different algorithms. In survival analysis, in many data sets, there is one favorite event and for each person, there is only one failure time and one cause of failure (type of event) (20). In some circumstances, it is possible that every subject experiences the event because on of k causes (k>2), called competing risks (21). For example, if we are interested in the analysis of time to death because of heart disease, factors other than heart disease that result in death are called competing risks. Hence, in competing risks data, there are at least two causes for failure that compete with each other for happening. When an individual experiences an event other than the desired event, the probability of the desired event will change. Therefore, it is necessary to perform competing risks analysis (22).

Analysis of survival data of competing risks has recently shown advantages over standard survival analyses. Regression competing risks modeling allows identification of independent risk factors (23).

### Statistical Analysis

In this study, continuous variables were presented as mean ± Standard Deviation. Cox proportional hazards regression was used to examine the relationship between the potential risk factors and all-cause mortality. In addition, competing risks model (24) was used to evaluate the association between the predictive variables and cause-specific (i.e., cardiac and vascular) mortality. In this study, time-to-death (month) was the primary outcome variable and *P*<0.05 was considered statistically significant. Data analyses were performed using Stata software package, ver. 13.

## Results

The patients’ mean ± SD age was 66.32±17.92 yr (65.01±19.98 yr in males vs. 67.42±15.78 yr in females) (*P*<0.001). According to Table 1, among the study patients, 52.3% were female and 47.6% were male. Baseline characteristics of the participants have been presented in Table 1.

**Table 1:** Baseline characteristics of the study subjects

**Variables**		**Frequency**	**Percent**	***P*-value**
Sex	Male	429	47.6	0.149
	Female	472	52.3	
Diabetes mellitus	Yes	107	11.86	0.001
	No	795	88.14	
Obesity	Yes	1	0.02	0.001
	No	901	99.98	
Cerebrovascular accident	Yes	39	4.32	0.001
	No	863	95.68	
Cardiomegaly	Yes	4	0.04	0.001
	No	898	99.96	
Smoking	Yes	128	14.19	0.001
	No	774	85.81	
Hypertension	Yes	353	39.13	0.001
	No	549	60.87	
Ischemic heart disease	Yes	283	31.37	0.001
	No	619	68.62	
Congenital heart disease	Yes	9	1.00	0.001
	No	893	99.00	
Valvular heart disease	Yes	194	21.51	0.001
	No	708	78.49	
Cardiomyopathy	Yes	10	1.11	0.001
	No	892	98.89	
Syncope	Yes	105	11.64	0.001
	No	797	88.36	
Atrioventricular block	Yes	566	62.75	0.001
	No	336	37.25	
Sick sinus syndrome	Yes	92	10.20	0.001
	No	810	89.80	
Atrial fibrillation	Yes	17	1.88	0.001
	No	885	98.12	

Until Mar 2012, 252 deaths (20.88%) were reported out of which, 46 (18.25%) were cardiac and 16 (6.35%) were vascular. In addition, 190 deaths (75.40%) were due to other causes. Totally, 955 cases were censored. The patients’ mean and median survival times were 50.58+35.61 and 51 months, respectively. Besides, the mean survival times of the patients with single-chamber and two-chamber pacemakers were 54.40±34.62 and 49.69±34.95 months, respectively.

### All-cause and cause-specific mortality

According to Table 2, age (HR=1.01, 95% CI: 1.00–1.02) and sick sinus syndrome (HR=1.65, 95% CI: 1.11–2.46) increased the risk of all-cause mortality, while two-chamber pacemaker (HR=0.68, 95% CI: 0.49–0.95) decreased the risk of all-cause mortality.

**Table 2:** The relationship between the study factors and all-cause / cause specific mortality

**Univariate analysis Variables**	**Cox regression All-cause death HR (95% CI)**	**Competing risk**	
		**Cardiac death SHR (95% CI)**	**Vascular death SHR (95% CI)**
Age	1.01 (1.00;1.02)^*^	1.03 (0.99;1.07)	1.03 (0.97;1.08)
Sex	0.95 (0.72;1.24)	0.58 (0.301.15)	0.47 (0.16;1.42)
Diabetes mellitus	1.15 (0.77;1.70)	0.93 (0.332.64)	7.15 (4.0820.12)^*^
Blood sugar	1.00 (1.00;1.00)	1.00 (0.99;1.01)	0.99 (0.95;1.03)
Obesity	3.00 (0.42;21.42)	21.83 (2.87;166.10)^*^	-
Cerebrovascular accident	0.95 (0.45;2.02)	1.71 (0.40;7.25)	2.06 (0.27;15.77)
Cardiomegaly	1.68 (0.24;12.03)	-	-
Creatinine	1.11 (0.89;1.37)	1.22 (0.89;1.67)	1.00 (0.63;1.60)
Smoking	0.92 (0.62;1.39)	1.66 (0.72;3.81)	1.06 (0.23;4.81)
Hypertension	1.07 (0.81;1.41)	1.28 (0.66;2.50)	0.57 (0.18;1.82)
Systolic blood pressure	1.00 (0.99;1.00)	0.99 (0.98;1.01)	0.99 (0.96;1.02)
Diastolic blood pressure	0.99 (0.98;1.00)	0.99 (0.97;1.03)	0.99 (0.93;1.05)
Ischemic heart disease	1.01 (0.75;1.34)	1.49 (0.75;2.93)	0.83 (0.26;2.66)
Congenital heart disease	1.25 (0.31;5.02)	3.57 (0.48;26.39)	-
Valvular heart disease	1.10 (0.79;1.52)	1.83 (0.89;3.77)	2.90 (1.01;8.29)^*^
Cardiomyopathy	0.91 (0.23;3.68)	-	-
Syncope	0.78 (0.50;1.22)	0.94 (0.33;2.71)	1.20 (0.27;5.45)
Atrioventricular block	1.09 (0.82;1.44)	0.83 (0.42;1.63)	0.66 (0.23;1.86)
Sick sinus syndrome	1.65 (1.11;2.46)^*^	0.96 (0.29;3.13)	1.67 (0.37;7.59)
Atrial fibrillation	0.91 (0.34;2.46)	1.38 (0.18;10.33)	-
Pacemaker	0.68 (0.49;0.95)^*^	0.51 (0.22;1.21)	0.38 (0.08;1.78)

* *P*<0.05, 1 cardiac death, 2 vascular death, 3 other deaths

**Fig. 1:**
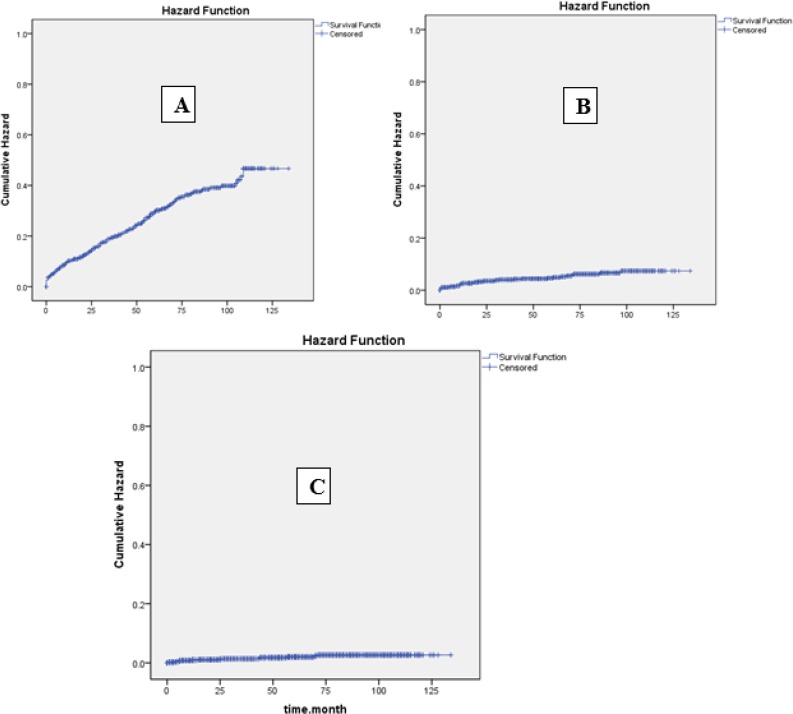
Comparison of cumulative incidence rates of mortality: A) all-cause, B) cardiac, and C) vascular death

The results also showed that obesity (HR=21.83, 95% CI: 2.87–166.10) increased the risk of cardiac death, while valvular heart disease (HR=2.90, 95% CI: 1.01–8.29) and diabetes mellitus (HR=7.15, 95% CI: 4.08–20.12) increased the risk of vascular death (Fig.1).

## Discussion

Assessment of the relationship between exposure and favorite event in the presence of competing risks is of one of the advanced aspects of survival analysis.

Moreover, the factors affecting the prognosis of death are very important in patients with artificial cardiac pacemaker implantation.

In this retrospective cohort study, the following results were obtained. Firstly, a significant relationship was found between age and all-cause mortality, such a way that older groups had a greater risk of death. This was consistent with the findings of the other studies (25, 26). As expected, age was an independent prognostic factor, with every year increasing the risk of death by 5% (1). A 9% (univariate) increase was reported in mortality in the subgroup of older patients (2). Yet, future studies with larger sample sizes are necessary to investigate the long-term survival after pacemaker implantation in different age groups, especially children.

Secondly, the present study results showed a significant relationship between sick sinus syndrome and all-cause mortality. Besides, a significant relationship was found between sick sinus syndrome and all-cause death (2).

Thirdly, single-chamber pacemaker had an adverse effect on all-cause death. A retrospective study was conducted on short-term survival with a 2-year follow-up and revealed that after adjusting for other factors, single-chamber pacemaker had an adverse effect on all-cause death (27). Similar results were also obtained in another study (2). Interestingly, the results of the MOST study demonstrated that the incidence of heart failure was higher in patients with single-chamber pacemakers compared to those with two-chamber pacemakers (28). This might also account for the difference in survival time in our study.

Fourthly, the current study findings disclosed a significant association between obesity and increased risk of cardiac mortality. Obesity is a risk factor for diabetes, hypertension, and dyslipidemia, which are risk factors for heart disease (29). Valvular heart disease and diabetes mellitus were associated with increased risk of vascular death. The present study also aimed to provide a risk model for predicting death after permanent pacemaker implantation. Several studies have investigated long-term survival after pacemaker implantation using Kaplan–Meier method (2, 4–7). However, Kaplan–Meier estimates cannot be assumed as probabilities when competing risks are present.

Overall, the results and conclusions should be investigated with caution. Retrospective study design can result in bias. In this research, all the information was gathered from the patients’ medical records. Although much work was done on these data to change them into the standard format, most of the information was qualitative. In addition, there were a limited number of obese subjects, resulting in low statistical power. Thus, the findings related to the obese individuals should be interpreted with caution. When statistical analysis was done between sub-groups, a type II error may occur. Hence, our results might have been influenced by residual confoundings, such as varying types of single-chamber pacemakers, not measured in this study. Moreover, no data was available about the variables not recorded in the patients’ medical records. Finally, the cause of mortality was determined by the registration system of the Department of Health of Shiraz University of Medical Sciences and the death certificates might have been biased by the choices of the physicians who filled them out. On the other hand, the strengths of this study included its representative population, relatively large sample size, and long-term follow-up. In addition, a unique aspect of this study was the ability to differentiate between the causes of death.

## Conclusion

The variables predicting mortality in the all-cause model were completely different from those in the cause-specific model. Moreover, studies performed on pacemaker up to now have used survival analysis, while death in such patients may occur due to reasons other than pacemaker. Therefore, future studies, particularly prospective ones, are recommended to use competing risk models.

## Ethical considerations

Ethical issues (Including plagiarism, informed consent, misconduct, data fabrication and/or falsification, double publication and/or submission, redundancy, etc.) have been completely observed by the authors.
